# Role of an Indole-Thiazolidine Molecule PPAR Pan-Agonist and COX Inhibitor on Inflammation and Microcirculatory Damage in Acute Gastric Lesions

**DOI:** 10.1371/journal.pone.0076894

**Published:** 2013-10-04

**Authors:** José Roberto Santin, Isabel Daufenback Machado, Stephen F. P. Rodrigues, Simone Teixeira, Marcelo N. Muscará, Suely Lins Galdino, Ivan da Rocha Pitta, Sandra H. P. Farsky

**Affiliations:** 1 Laboratory of Experimental Toxicology, Department of Clinical and Toxicological Analyses, School of Pharmaceutical Sciences, University of Sao Paulo, Sao Paulo, Brazil; 2 Department of Pharmacology, Institute of Biomedical Sciences, University of São Paulo, São Paulo, Brazil; 3 Department of Chemistry, Federal University of Pernambuco, Pernabumbuco, Recife, Brazil; Centro di Riferimento Oncologico, IRCCS National Cancer Institute, Italy

## Abstract

The present study aimed to show the *in vivo* mechanisms of action of an indole-thiazolidine molecule peroxisome-proliferator activated receptor pan-agonist (PPAR pan) and cyclooxygenase (COX) inhibitor, LYSO-7, in an ethanol/HCl-induced (Et/HCl) gastric lesion model. Swiss male mice were treated with vehicle, LYSO-7 or Bezafibrate (p.o.) 1 hour before oral administration of Et/HCl (60%/0.03M). In another set of assays, animals were injected i.p. with an anti-granulocyte antibody, GW9962 or L-NG-nitroarginine methyl ester (L-NAME) before treatment. One hour after Et/HCl administration, neutrophils were quantified in the blood and bone marrow and the gastric microcirculatory network was studied *in situ*. The gastric tissue was used to quantify the percentage of damaged area, as well as myeloperoxidase (MPO), inducible nitric oxide synthase (iNOS), endothelial nitric oxide synthase (eNOS) protein and PPARγ protein and gene expression. Acid secretion was evaluated by the pylorus ligation model. LYSO-7 or Bezafibrate treatment reduced the necrotic area. LYSO-7 treatment enhanced PPARγ gene and protein expression in the stomach, and impaired local neutrophil influx and stasis of the microcirculatory network caused by Et/HCl administration. The effect seemed to be due to PPARγ agonist activity, as the LYSO-7 effect was abolished in GW9962 pre-treated mice. The reversal of microcirculatory stasis, but not neutrophil influx, was mediated by nitric oxide (NO), as L-NAME pre-treatment abolished the LYSO-7-mediated reestablishment of microcirculatory blood flow. This effect may depend on enhanced eNOS protein expression in injured gastric tissue. The pH and concentration of H^+^ in the stomach were not modified by LYSO-7 treatment. In addition, LYSO-7 may induce less toxicity, as 28 days of oral treatment did not induce weight loss, as detected in pioglitazone treated mice. Thus, we show that LYSO-7 may be an effective treatment for gastric lesions by controlling neutrophil influx and microcirculatory blood flow mediated by NO.

## Introduction

Gastric ulcers, a chronic disease that affects millions of people worldwide, are considered a global health problem and are linked to gastric cancer [1,2]. The genesis of the disease is associated with an imbalance of endogenous protective agents and aggressors to the gastric mucosa. Moreover, exogenous factors, such as stress and the excessive intake of non-steroidal anti-inflammatory drugs (NSAIDs) or alcohol may also cause gastric injury. Experimental models using ethanol/HCl (Et/HCl) to induce gastric ulcers have been recently employed to study the genesis and progression of gastric lesions and to evaluate therapeutic approaches [3–7]. Alcohol intake causes mucosal edema, hemorrhage, sub-epithelial cell exfoliation, and infiltration of inflammatory cells. These latter cells release chemical mediators, which contribute to vasoconstriction/ischemia and stasis in the microcirculation, leading to the death of gastric cells [8–10].

Nitric oxide (NO) produced in the stomach is strongly involved in inflammatory and vascular processes, and may exert both protective and deleterious effects during gastric ulceration [11]. Endothelial nitric oxide synthase (eNOS)-produced NO exerts protective actions by increasing vasodilatation and blood flow, and also reduces endothelial leukocyte interactions in the microcirculation [12]. Furthermore, expression and activation of inducible nitric oxide synthase (iNOS) by constitutive and recruited cells during gastric ulceration leads to increased NO expression, which enhances tissue injury by evoking inflammatory cell infiltration and edema formation. Thus, the balance between iNOS/eNOS expression and activity in gastric tissue is important for adequate production of NO, which contributes to the maintenance of the structure of gastric tissue in response to aggressive agents [13,14].

The main currently available gastric ulcer treatment involves oral administration of histamine receptor antagonists (H2-Ras) and/or proton pump inhibitors (PIPs), which act by inhibiting gastric secretion. As gastric ulcers are caused by a complex mechanism, the search for new therapeutic pathways has been the target of recent investigations.

Peroxisome proliferator-activated receptor (PPAR) is a transcription factor of the nuclease hormone receptor superfamily. Three isoforms have been identified, i.e. PPARα, PPARβ/δ and PPARγ; they are broadly distributed in the organism and are responsible for regulating important physiological process. PPAR isoforms play key roles in the regulation of adipogenesis, insulin sensitivity and energy homeostasis [15,16]. Evidence has shown that PPAR regulates several actions of immune cells, exerting a marked effect on the control of inflammation. The mechanisms of action are complex and not yet completely understood, as post-transcriptional mechanisms have been proposed for the α and δ isoforms [17,18], and PPARγ activation can induce transactivation or transrepression of target genes [19–22]. PPAR pan-agonists, which activate all three PPAR isoforms, have been suggested as a potential tool as they amplify the therapeutic action and reduce the adverse effects [23,24].

Recent evidence has demonstrated that simultaneous activation of PPAR isoforms and inhibition of cyclooxygenase-2 (COX-2) may be a good approach to treat inflammatory diseases and cancer [25,26]. Despite the use of PPAR agonists in a diversity of inflammatory disorders, only little evidence has associated PPAR activation by thiazolidine molecules, such as pioglitazone and rosiglitazone, with the control and healing of gastric tissue damage. Lesions caused by ischemia/reperfusion or NSAID intake were reduced in rats pre-treated with a PPARγ agonist, and the beneficial effect was correlated to reduced mRNA levels and protein content of pro-inflammatory cytokines and enzymes, such as COX-2, iNOS, and oxidative enzymes, as well as overexpression of platelet-endothelial cell adhesion molecule (PECAM-1) and heat-shock protein 70 (HSP70) in injured gastric tissue [27–31]. More recently, it has been shown that rosiglitazone prevents indomethacin-induced gastric ulcers in type II diabetic rats [32].

Here, we investigated the efficacy and mechanisms of action of an indole-thiazolidine molecule designed to be a PPAR pan-agonist and COX inhibitor, named LYSO-7 [33], on Et/HCl-induced gastric lesions in mice. LYSO-7 provided cytoprotection by impairing neutrophil influx and reestablishing the vascular network. The latter effect was mediated by the *in vivo* balance of iNOS/eNOS protein expression. To our knowledge, the proposed mechanism of a PPAR pan-agonist molecule has not been previously demonstrated *in vivo* in an Et/HCl model, and points out the use of PPAR pan-agonists as a possible therapeutic approach for acute gastric lesions.

## Materials and Methods

### Animals

Male Swiss mice (20–30 g) were provided by the Central Animal House of the School of Pharmaceutical Science and the Chemistry Institute of the University of São Paulo. The animals were housed in standard cages, at room temperature (25±3°C), with 12 h dark/12 h light cycles, and supplemented with food and water *ad libitum*. They were transferred to the laboratory 12 hours prior to the experiments and were given water *ad libitum*. In all experiments, the animals were kept in cages with raised floors constructed from wide mesh, to prevent coprophagy.

### Ethics statement

The project was approved by the Institutional Animal Care and Use Committee (IACUC) at the School of Pharmaceutical Sciences, University of São Paulo (Protocol number: 298 and 399). All procedures were performed according to the Brazilian Society of Science of Laboratory Animals guidelines for the proper care and use of experimental animals.

### Drug treatments

Bezafibrate, pioglitazone (Sigma, St Louis, MO, USA) and LYSO-7 [33] were dissolved in water containing 0.5% (v/v) ethanol absolute, and then added to 2% carboxymethylcellulose (CMC) in distilled water as the vehicle. In some cases, before the treatments, the mice were additionally treated intraperitoneally with L-NG-nitroarginine methyl ester (L-NAME; Sigma, St Louis, MO, USA), anti-granulocyte antibody (eBioscience, San Diego, CA, USA) or GW9962 (Sigma, St Louis, MO, USA).

### Gastric lesion protocol

The experiment was carried out according to the method of Mizui and Doteuchi [34]. After 12 h of fasting, the mice were randomly divided into six groups of five animals and treated as follows: 1) naïve (untreated animals); 2) CMC 0.2% aqueous solution (vehicle, 100 µL/10 g body weight); 3) bezafibrate (25 mg/kg) 4) bezafibrate (50 mg/kg); 5) LYSO-7 (5 mg/kg); 6) LYSO-7 (25 mg/kg); 7) LYSO-7 (50 mg/kg). One hour after oral treatment with the above solutions, all animals received ethanol 60% + HCl 0.03 M (100 µL/10g body weight), a concentration considered relevant to alcohol ingestion in man [35], by the oral route, to induce gastric ulcers. One hour later, the animals were sacrificed, and the stomachs were removed and opened along the greater curvature. The stomachs were gently rinsed with water to remove the gastric contents and blood clots, and then scanned. The images obtained were analyzed using “EARP” software to measure each lesion.

### Determination of gastric secretion

Pylorus ligation is an important procedure that shows possible changes to gastric content parameters, e.g., total acidity and pH [36]. The assay employed here was based on a method described by Shay [37] with a few modifications. Briefly, the animals were divided into groups (n=5), according to the treatment used, as previously described. After 18 h of fasting, the animals were anesthetized; the abdomen was incised, and the pylorus ligated. Immediately after pylorus ligation, LYSO-7 was administered at a dose of 50 mg/kg. Omeprazole (30 mg/kg) was used as the positive control, and the vehicle was administered as the negative control. All the samples were administered intraduodenally. Four hours later, the animals were sacrificed; the abdomen was opened, and another ligature was placed at the esophageal end. The stomachs were removed, and the gastric contents were collected and centrifuged at 3,000 rpm (8,000×g, 25°C, 10 min). The amount of gastric juice acid (in milliliters) and the pH values were determined. The total acid secretion in the gastric juice was determined in the supernatant volume by titration (pH 7.0), using a 0.01 M NaOH solution and phenolphthalein as the indicator.

### GW9662 pre-treatment

To evaluate PPARγ agonist activity, animals were pre-treated with GW9662, an PPARγ antagonist or PBS, 30 min before treatment with LYSO-7; after 1 hour, the Et/HCl solution was administered. One hour later, the animals were sacrificed, and the stomachs were removed and opened along the greater curvature. The stomachs were gently rinsed with water to remove the gastric contents and blood clots, and then scanned. The images obtained were analyzed using “EARP” software to measure each lesion.

### Histological analysis

After macroscopic analysis, a small portion of each stomach was ﬁxed in 4% formalin solution, dehydrated through increasing grades of alcohol and embedded in parafﬁn. Five-micrometer sections were made using a microtome, and stained with hematoxylin–eosin solution. Tissue preparations were observed and micro-photographed using an Axioplan 2 Zeiss light microscope. Five different fields of the tissue were evaluated to count the number of leukocytes.

### Myeloperoxidase (MPO) assay

The activity of myeloperoxidase (MPO), a hemoprotein located in the azurophilic granules of neutrophils, was used as a biochemical marker for neutrophil inﬁltration into the studied tissues. MPO activity was measured according to the method originally described by Bradley [38] with some modiﬁcations, after heating the organ homogenates at 60°C for 2 h in order to inactivate endogenous catalase. Brieﬂy, after homogenizing the tissue samples in the presence of hexadecyl trimethyl ammonium bromide (HTAB, Sigma, St Louis, MO, USA) in order to disrupt the granules, the tubes were centrifuged at 10,000 x g for 5 min. MPO activity was analyzed in the supernatants by its capacity to catalyze the oxidation of o-dianisidine dihydrochloride (Sigma, St Louis, MO, USA) in the presence of hydrogen peroxide. Absorbance was monitored at 460 nm (Spectramax Plus 384, Molecular Devices Inc., Sunnyvale, USA) and the obtained V_max_ (maximum speed) parameter, related to enzyme activity, was determined using a molar extinction coefﬁcient of 11,300 M cm^-1^.

### Anti-granulocyte antibody treatment

To determine whether neutrophil depletion encompasses the entire period of neutrophil infiltration, test mice were injected with 50 µL (0.025 mg) of rat anti-mouse Ly6G (Gr-1) antibody, clone RB6-8C5 (eBioscience, San Diego, CA, USA), 48 h before the experiment. Previous findings showed that this Ly6G/Ly6C antibody specifically depletes mature granulocytes, especially neutrophils, and does not influence the total monocyte/macrophage count [39,40]. Control mice were injected with 50 µL of PBS. Neutrophil depletion was confirmed by optical microscopy.

### Intravital microscopy

Animals were anesthetized with ketamine:xylazine (0.2:0.02 g/kg body weight), injected intraperitoneally, and tracheostomized to facilitate respiration. The femoral vein was cannulated to inject the fluorescent agent rhodamine. Leukocytes were labeled with 0.02% rhodamine immediately before blood vessel visualization. A portion of the stomach was surgically exteriorized to allow observation of the microcirculation. Animals were maintained on a special board thermostatically controlled at 37°C. The preparation was kept moist and warmed by irrigating the tissue with a warmed (37°C) Tyrode’s solution (in mg/mL: 8 NaCl; 0.2 KCl; 0.1 MgCl_2_; 0.058 NaH_2_PO_4_.H_2_O; 1 glucose; 1 NaHCO_3_) (pH 7.2–7.4). The rate of solution flow onto the exposed tissue was controlled (2 mL/min) to keep the preparation in continuous contact with solution. Images were obtained by fluorescence microscopy (Carl-Zeiss, Axioplan II) with epi-illumination, captured with a video camera and simultaneously transmitted to a computer. Assays were performed with animals submitted to the Et/HCl-induced ulcer model, pre-treated or not with L-NAME, and treated or not treated with LYSO-7. Five different fields of the microcirculation were evaluated to determine the percentage of blood vessels in stasis.

### PPARγ mRNA expression

PPARγ mRNA transcripts were quantified in stomach tissue samples by real-time reverse transcription polymerase chain reaction (RT-PCR) analysis as previously reported. The samples were stored at −80°C in RNA-free Eppendorf tubes containing a lysis and nucleic acid purification solution (Lysis Solution 2X), homogenized by centrifugation and then digested with proteinase K (200 µg mL^−1^ in lysis solution) overnight at room temperature. Samples were centrifuged at 14,000 rpm for 5 min and the aqueous phase containing RNA was recovered. Total RNA extraction was performed with an ABI PRISM 6100 Nucleic Acid Prepstation (Applied Biosystems, USA) and its related chemistry. RNA was dried using a UNIVAPO 100H drier (UNIEQUIP). Subsequently, the RNA was dissolved in RNAse-free water (DEPC water) and quantified with a Nanodrop spectrophotometer (ND-420, Nanodrop Technology). Nucleic acid quality was assessed by measuring the A260/A280 ratio. Ten microliters of total RNA were used for the RT reaction following the manufacturer’s protocol (High Capacity cDNA Archive kit, Applied Biosystems, USA). The cDNA was then amplified by real-time PCR. The reaction mix (all reagents from Applied Biosystems) contained TaqMan DNA polymerase (TaqMan® Universal PCR Master Mix 2X) and primers. Real-time PCR was performed with an ABI PRISM 7000 Sequence Detection System (Applied Biosystems, USA).

### iNOS, eNOS and PPARγ protein expression

iNOS, eNOS and PPARγ protein expression in the stomach was quantified by Western blot. Briefly, stomach tissue proteins were extracted in Tris buffer (50 mM, pH 7.4) containing leupeptin (10 µg/mL), soybean trypsin inhibitor (10 µg/ml), aprotinin (2 µg/ml) and PMSF (1 mM). Homogenized proteins (87.5 µg) were separated by sodium dodecyl sulfate-polyacrylamide gel electrophoresis (SDS–PAGE; 7%) and were electrophoretically transferred to a nitrocellulose membrane. After blocking non-specific sites with 1% casein, membranes were incubated overnight with primary rabbit polyclonal antibodies raised against iNOS (BD, CA, USA), eNOS (BD, CA, USA) and PPARγ (Santa Cruz Biotechnology, INC, CA, USA) (500 ng/mL). Membranes were washed with Tris-buffered saline containing 0.1% Tween-20 and incubated with horseradish peroxidase-conjugated goat anti-rabbit secondary antibody. A chemiluminescent assay (HRP SuperSignalWestPico; Pierce, USA) was used to detect immunoreactive bands. The intensities of the bands were estimated by densitometry analysis and were compared to the intensity of β-actin expression.

### Effects of LYSO-7 chronic treatment on body weight

To evaluate possible adverse effects associated with LYSO-7 administration (50 mg/kg, oral route), the animals were treated daily for 28 days by the oral route. Body weight and food consumption were assessed every two days. Animals treated with pioglitazone (40 mg/kg, oral route) were used as the control.

### Statistical analysis

The means and standard error of the mean (SEM) of all data presented here were compared using Student’s t-test or ANOVA. Tukey’s multiple comparisons test and Dunnett’s test was used to determine the signiﬁcance of differences between values according to the experimental conditions. The statistical software GraphPad Prism® was used for this purpose. p<0.05 was considered signiﬁcant.

## Results

### In vivo LYSO-7 treatment protects Et/HCl-induced gastric tissue damage

**Figure 1:**
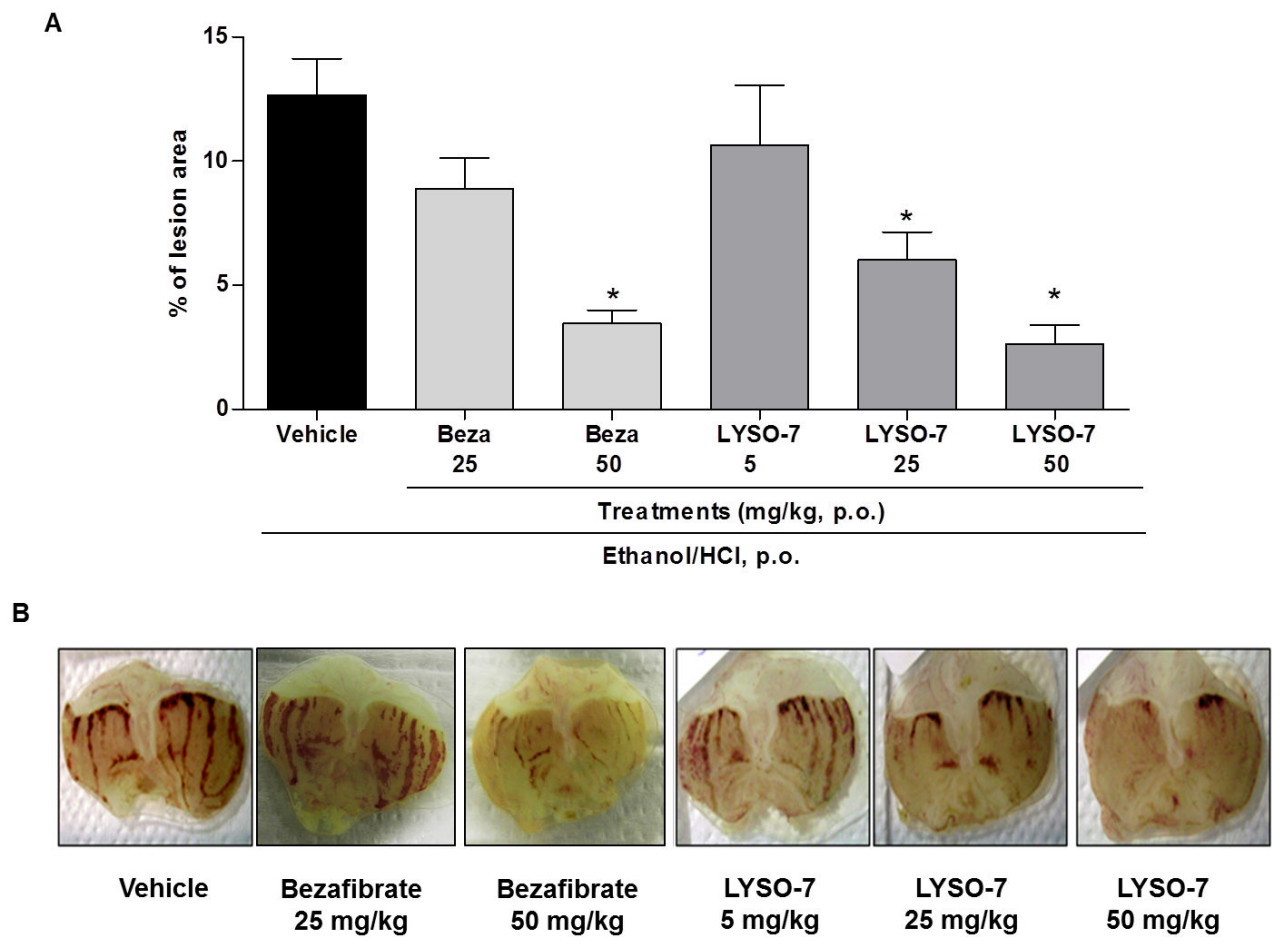
Effect of LYSO-7 on Et/HCl-induced gastric tissue damage. Male Swiss mice were treated with CMC (vehicle), LYSO-7 or bezafibrate, p.o., 1 hour before oral administration of Et/HCl solution. Gastric tissue was collected 1 hour after Et/HCl administration. (A) shows the percentage of the lesioned area and (B) shows representative images of the gastric tissue. Results are expressed as mean±SEM of 5 animals in each group. Statistical analysis was performed using ANOVA followed by Dunnett’s test. *P<0.01 vs. vehicle.

Oral administration of Et/HCl solution caused lesions in the gastric tissue, which were prevented in a dose-dependent manner by LYSO-7 pre-treatment (Figure 1). It is noteworthy that tissue protection was seen with 25 mg/kg of LYSO-7 and with 50 mg/kg of bezafibrate, a PPAR pan-agonist (Figure 1).

### In vivo LYSO-7 treatment enhances PPARγ gene and protein expression in the gastric tissue

**Figure 2:**
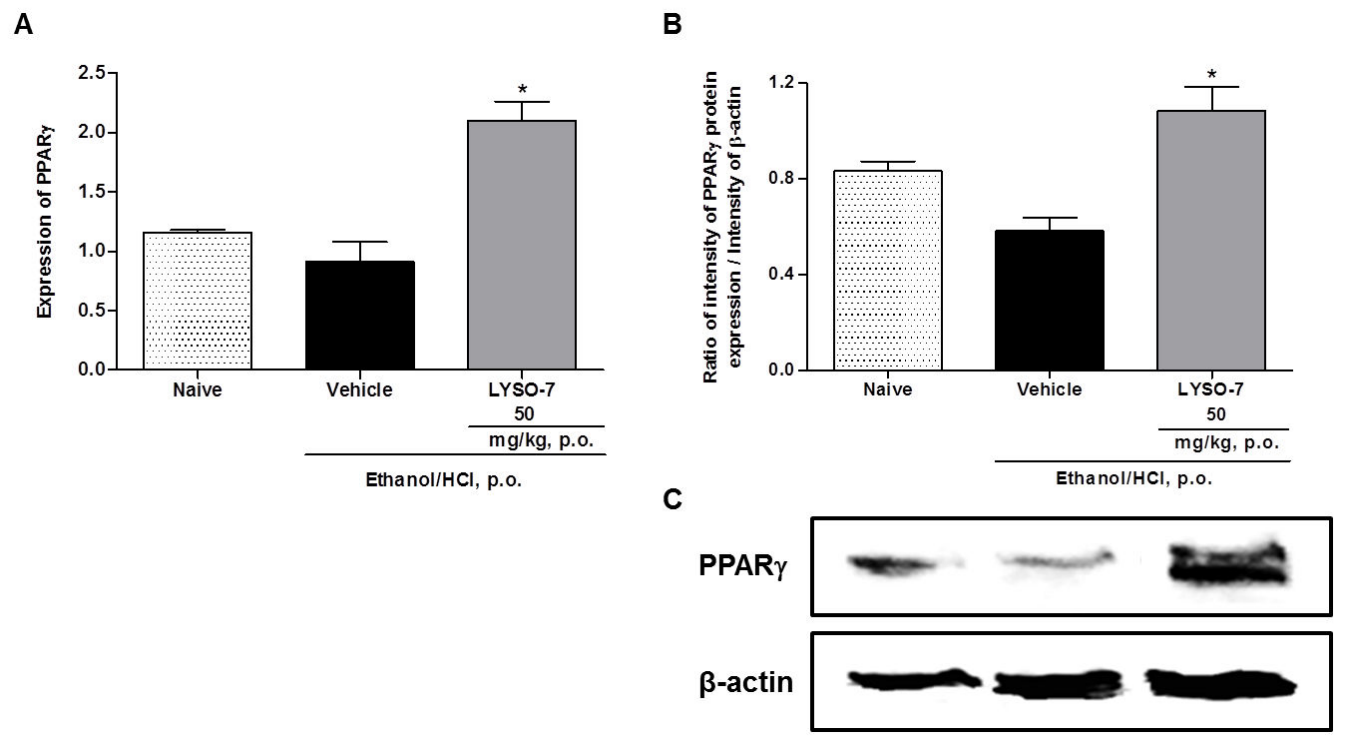
Effects of LYSO-7 treatment on PPARγ gene and protein expression in Et/HCl-damaged gastric tissue. Male Swiss mice were treated with CMC (vehicle) or LYSO-7, p.o., 1 hour before oral administration of Et/HCl solution, and gastric tissue was collected 1 hour later. (A) PPARγ gene expression and (B and C) PPARγ protein expression. Results are expressed as mean±SEM of 4 animals in each group. Statistical analysis was performed using ANOVA followed by Tukey’s test. *P<0.05 vs. vehicle.

**Figure 3:**
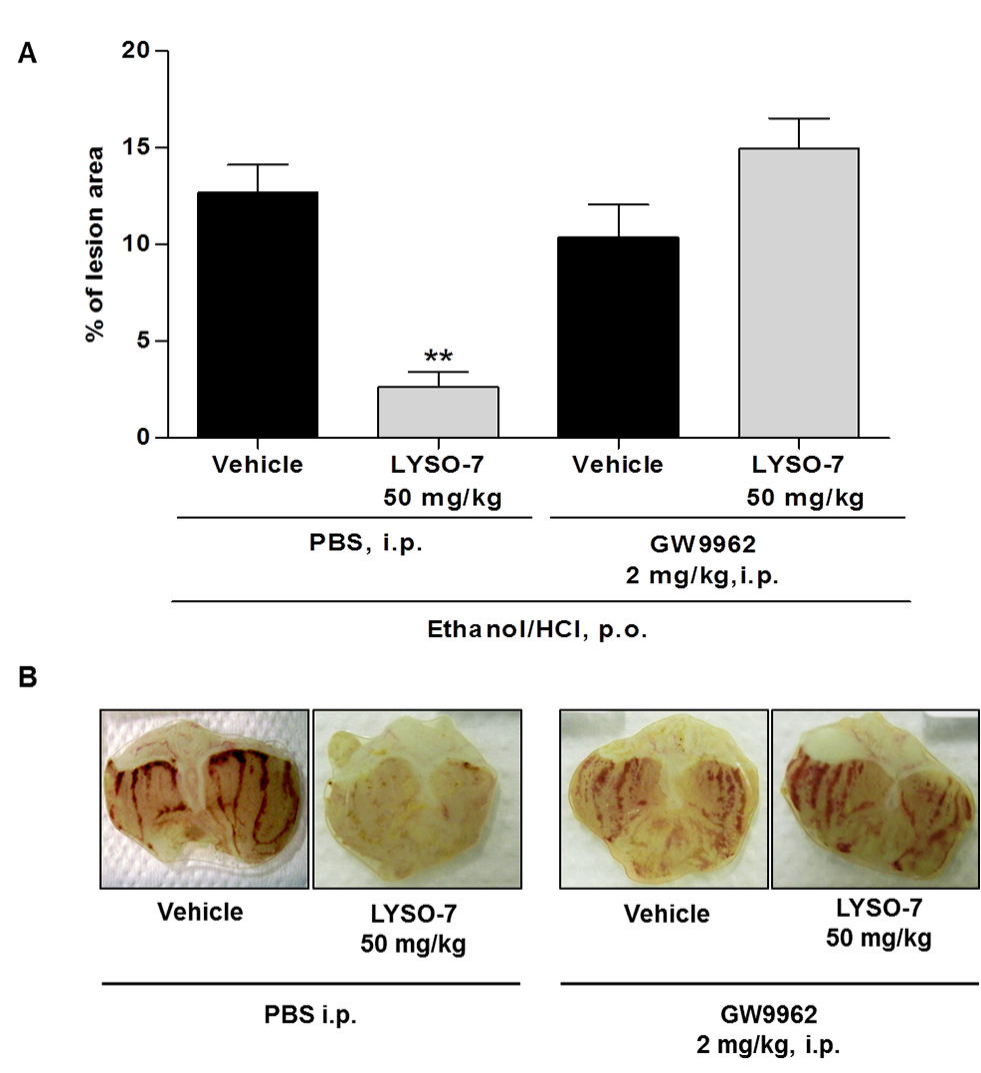
Role of PPARγ receptor in the protective effect of LYSO-7 on Et/HCl-induced gastric tissue damage. Male Swiss mice were pretreated with GW9962 or PBS (i.p.) and treated with CMC (vehicle) or LYSO-7 20 min later. Et/HCl solution was administered 1 hour after the treatments. Gastric tissue was collected 1 hour later. (A) shows the percentage of the lesioned area; (B) shows representative images of the gastric tissue. Results are expressed as mean±SEM of 5 animals in each group. Statistical analysis was performed using ANOVA followed by Dunnett’s test **P<0.01 vs. vehicle.

Tissue PPAR expression has been highlighted as a mechanism of action of PPAR agonists. Here, we show that this is a mechanism of action of LYSO-7, as levels of PPAR mRNA and protein in the gastric tissue were markedly enhanced after *in vivo* LYSO-7 treatment (Figure 2). In addition, we show that the effect of LYSO-7 in Et/HCl-induced gastric lesions is dependent on its PPARγ agonist activity, as the protective effect of LYSO-7 in gastric tissue was reversed in mice pre-treated with GW9962, a recognized antagonist of PPARγ (Figure 3A and B).

### LYSO-7 does not impair acid gastric secretion

Data presented in Table 1 show that the pH and H^+^ concentration in the stomach after pylorus ligation surgery were 3.26 and 135.0, respectively. These values were modified by omeprazole treatment, represented by increased and reduced pH and H^+^ concentration, respectively. On the other hand, LYSO-7 treatment did not affect gastric secretion parameters.

**Table 1:** Effects of LYSO-7 and omeprazole treatment on biochemical parameters of gastric juice obtained from mice with pylorus ligation.

Treatment	Dose	pH	[H+] mequiv./L/4h
	(mg/kg)		
Vehicle	-	3.26 ± 0.12	135.0 ± 10.41
Omeprazole	30	4.24 ± 0.19*	62.5 ± 5.18*
LYSO-7	50	3.32 ± 0.17	117.5 ± 18.33

Results are mean±SEM of 5 mice in each group. Statistical analysis was performed using ANOVA followed by Dunnett’s test *p<0.05 vs. vehicle

### LYSO-7 inhibits neutrophil migration into Et/HCl-damaged gastric tissue

**Figure 4:**
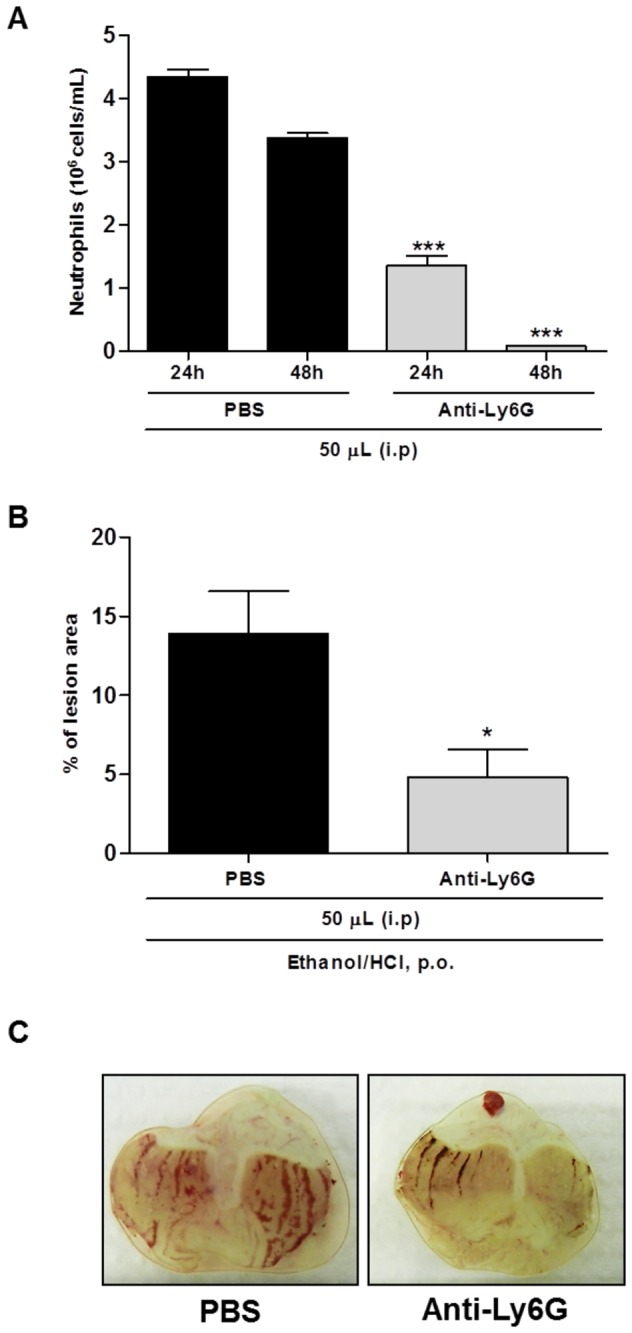
Role of neutrophils in Et/HCl-damaged gastric tissue. Male Swiss mice were pre-treated with PBS or anti-granulocyte antibody (i.p.) and blood was collected 24 or 48 hours later; Et/HCl solution was orally administered a further 48 hours later. Gastric tissue was collected 1 hour after Et/HCl delivery. (A) number of neutrophils in the blood; (B) percentage of the gastric lesion and (C) representative images of gastric tissue. Results are expressed as mean±SEM of 5 animals in each group. Statistical analysis was performed using ANOVA followed by Tukey’s test. *P<0.05 and ***P<0.001 vs. PBS treatment.

**Figure 5:**
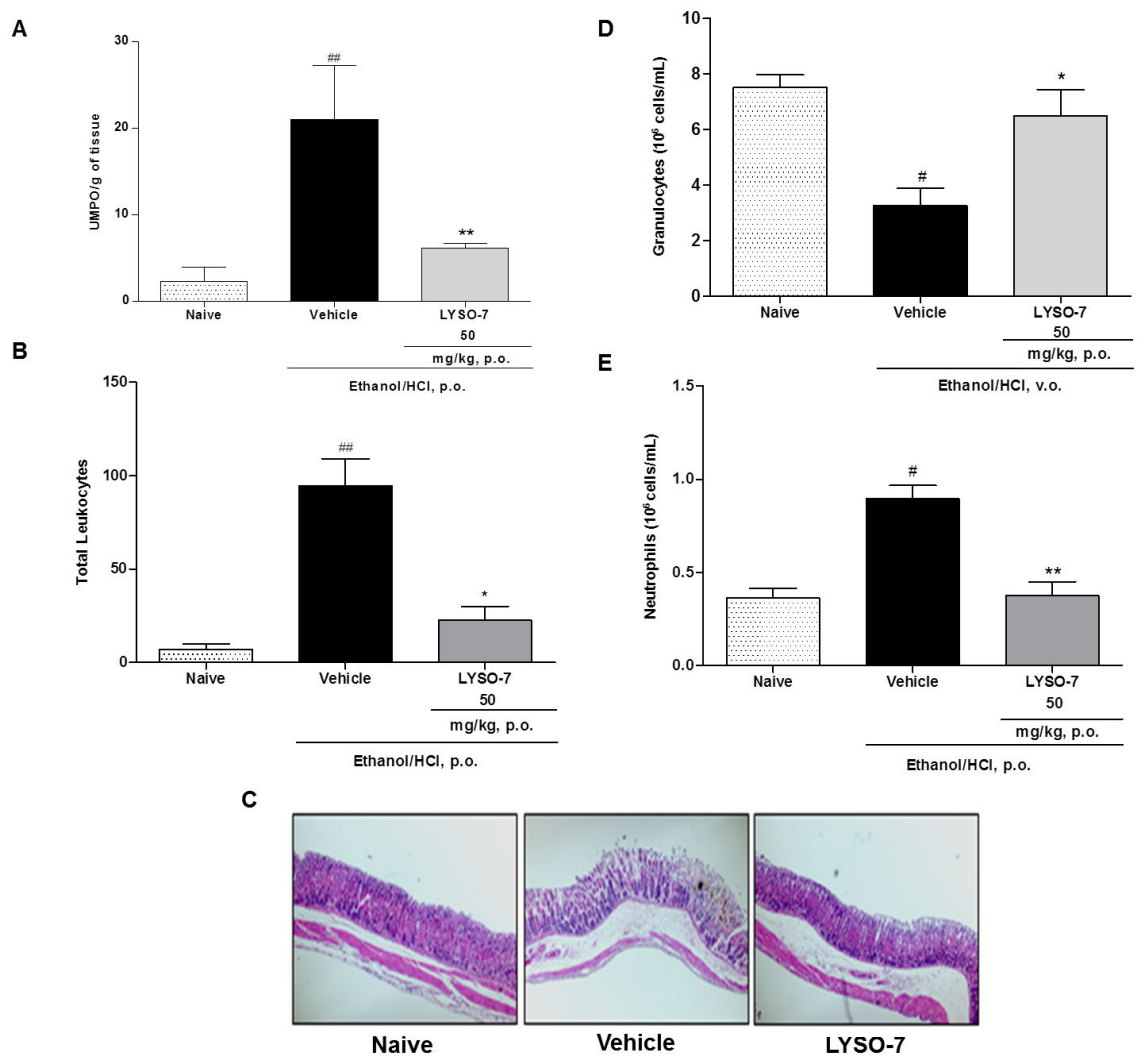
Effects of LYSO-7 on neutrophil influx into Et/HCl-damaged gastric tissue. Male Swiss mice were treated with CMC (vehicle) or LYSO-7, p.o., 1 hour before oral administration of Et/HCl solution and gastric tissue, blood and bone marrow perfusate were collected 1 hour later. (A) MPO activity in the gastric tissue; (B) leukocyte numbers in the gastric tissue; (C) representative image of leukocyte infiltration in the gastric tissue; (D) numbers of granulocytes in the bone marrow; (E) number of circulating neutrophils. Results are expressed as mean±SEM of 5 animals in each group. Statistical analysis was performed using ANOVA followed by Tukey’s test. *P<0.05; **P<0.01 vs. vehicle; ^#^ P<0.05; ^# #^ P<0.01 vs. naïve.

Although neutrophil participation in Et/HCl-induced gastric damage has been proposed, its actual role in the process has not been previously shown. Here, we show that neutrophil is a hallmark of the lesion, as administration of an anti-granulocyte monoclonal antibody into mice markedly reduced both the number of granulocytes in the blood (Figure 4A) and in the gastric lesion 48 hours later (Figure 4B and C). Moreover, histological images and enhanced MPO activity in the gastric tissue showed that neutrophils rapidly accumulated in the tissue after Et/HCl administration, which were reduced by LYSO-7 treatment (Figure 5A, B and C). LYSO-7 acts by blocking neutrophil trafficking from the bone marrow, as a reduced number of circulating neutrophils and enhanced number of mature granulocytes in the bone marrow were observed in LYSO-7 treated mice (Figure 5 D and E).

### LYSO-7 reverses blood flow stasis in Et/HCl-damaged gastric tissue, mediated by NO

**Figure 6:**
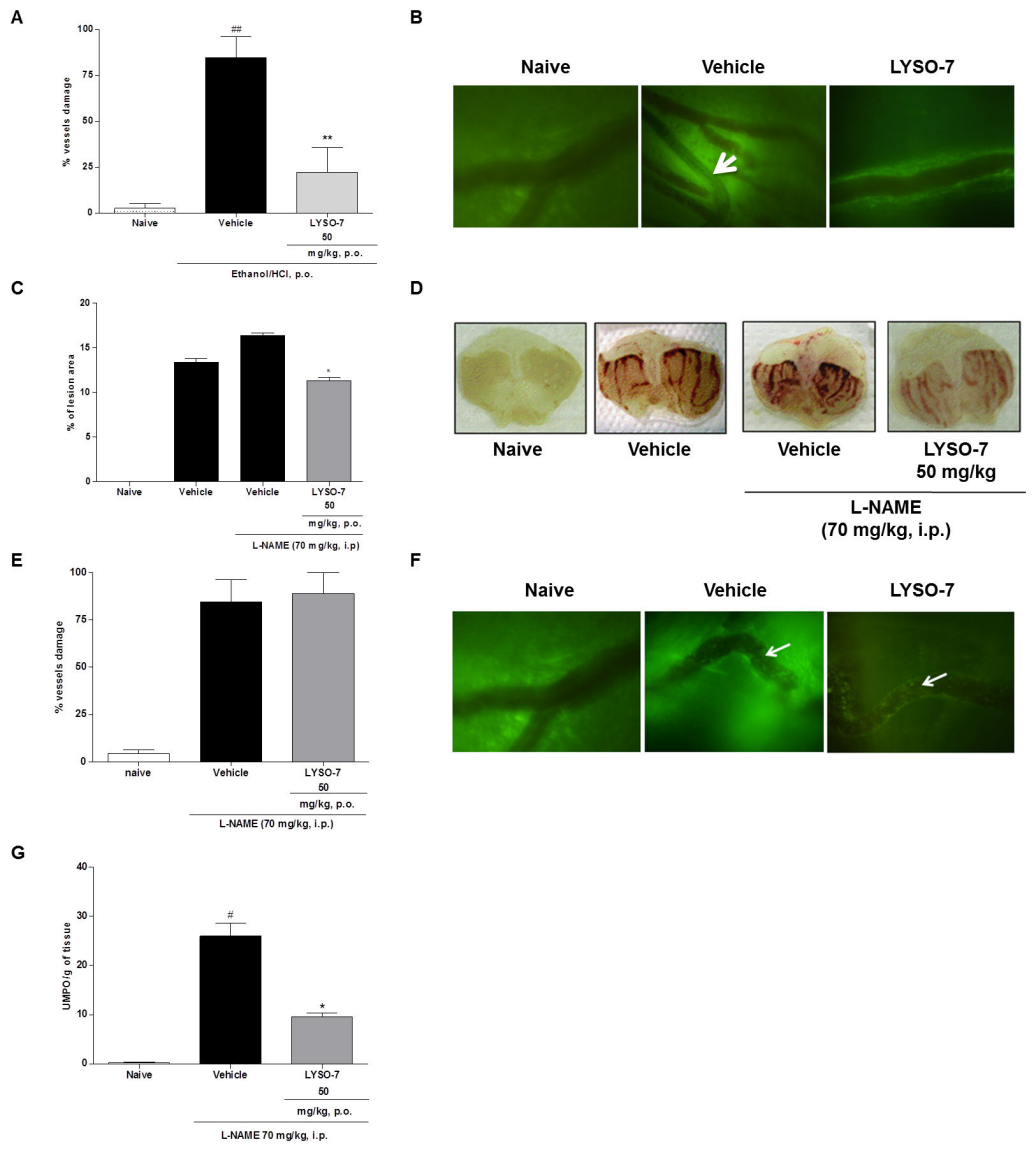
Effects of LYSO-7 on microcirculatory blood flow in Et/HCl-damaged gastric tissue. Male Swiss mice were pre-treated with L-NAME (i.p.) or treated with CMC (vehicle) or LYSO-7, p.o., 1 hour before oral administration of Et/HCl solution. Gastric tissue was surgically exposed 1 hour later after treatments for observation by intravital microscopy. (A) percentage of vessels in stasis; (B) representative images of microcirculatory vessels in the gastric tissue; (C) percentage of lesion area; (D) representative images of gastric tissue; (E) percentage of vessels in stasis; (F) representative images of microcirculatory vessels in the gastric tissue (G) MPO activity in gastric tissue. White arrows indicate vessels in stasis. Results are expressed as mean±SEM of 5 animals in each group. Statistical analysis was performed using ANOVA followed by Tukey’s test. **P<0.01 vs. vehicle; *P<0.05 vs. vehicle + L-NAME and ^# #^ P<0.01 vs. naïve.

The real-time intravital microscopy on the whole stomach carried out in this study is different than methods previously reported in the literature [41], as it does not involve surgical manipulation, but only exteriorization of the tissue. The data obtained here show that the Et/HCl administration caused rapid stasis in the microcirculatory network (Figure 6 A and B), and LYSO-7 pre-treatment prohibited vessel stasis (Figure 6 A and B)*.*

NO has been proposed as protective endogenous mediator of gastric damage induced by different agents. Here, we show that pre-treatment with L-NAME, a non-specific inhibitor of NO synthesis, enhanced gastric lesions caused by Et/HCl administration and partially inhibited the protective effect of LYSO-7 (Figure 6C and D). Furthermore, L-NAME treatment blocked the action of LYSO-7 on gastric microcirculatory blood flow (Figure 6E and F), but did not alter the reduced neutrophil influx into the gastric area caused by LYSO-7 (Figure 6 G).

LYSO-7 treatment inhibits iNOS expression and enhances eNOS protein expression in Et/HCl-damaged gastric tissue

**Figure 7:**
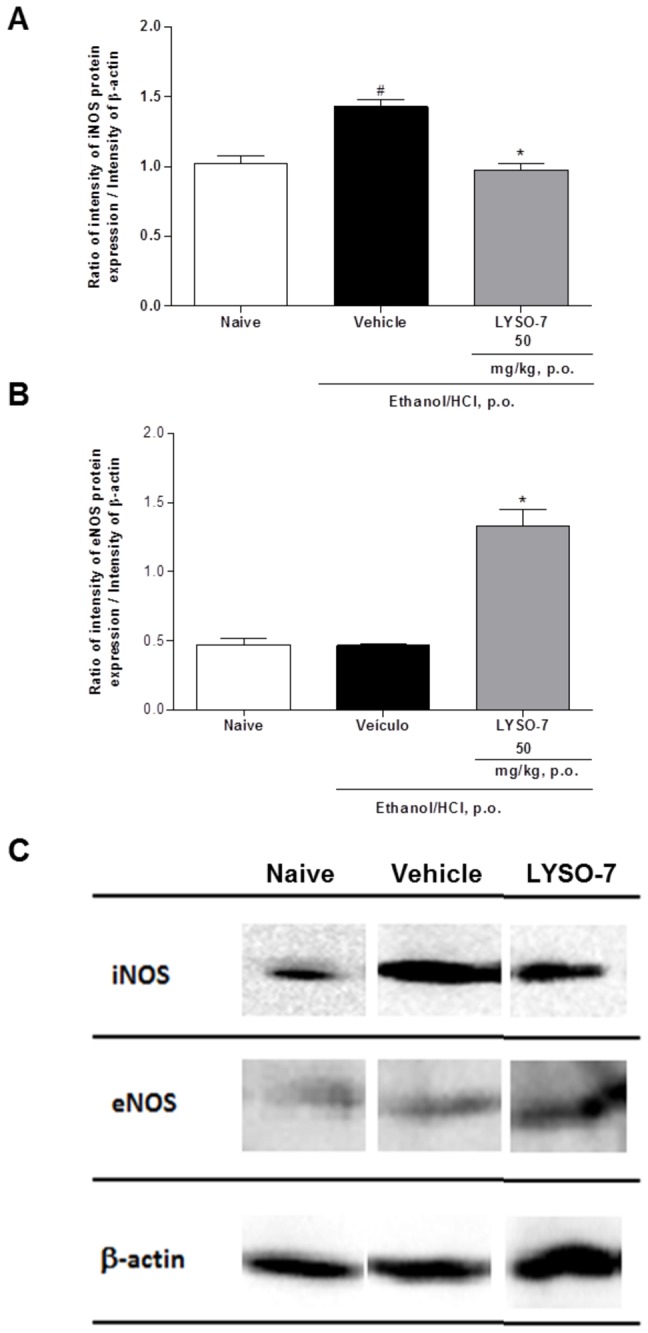
Effects of LYSO-7 on NOS expression in Et/HCl-damaged gastric tissue. Male Swiss mice were treated with CMC (vehicle) or LYSO-7, p.o., 1 hour before oral administration of Et/HCl solution and gastric tissue was collected 1 hour later. (A) iNOS; (B) eNOS protein expression on gastric tissue (C) representative image of gel electrophoresis. Results are expressed as mean±SEM of 4 animals in each group. Statistical analysis was performed using ANOVA followed by Tukey’s test. *P<0.05 vs. vehicle and ^#^ p<0.05 vs. naïve.

Based on the evidence supporting a role for NO in the protective effects of LYSO-7 in Et/HCl-damaged gastric tissue, and since eNOS/iNOS imbalance is involved in gastric ulcer disease, the actions of LYSO-7 on the protein expression of NOS enzymes were investigated in gastric tissue. The data presented in Figure 7 show that LYSO-7 inhibited iNOS and enhanced eNOS protein expression.

### LYSO-7 treatment does not alter body weight

**Figure 8:**
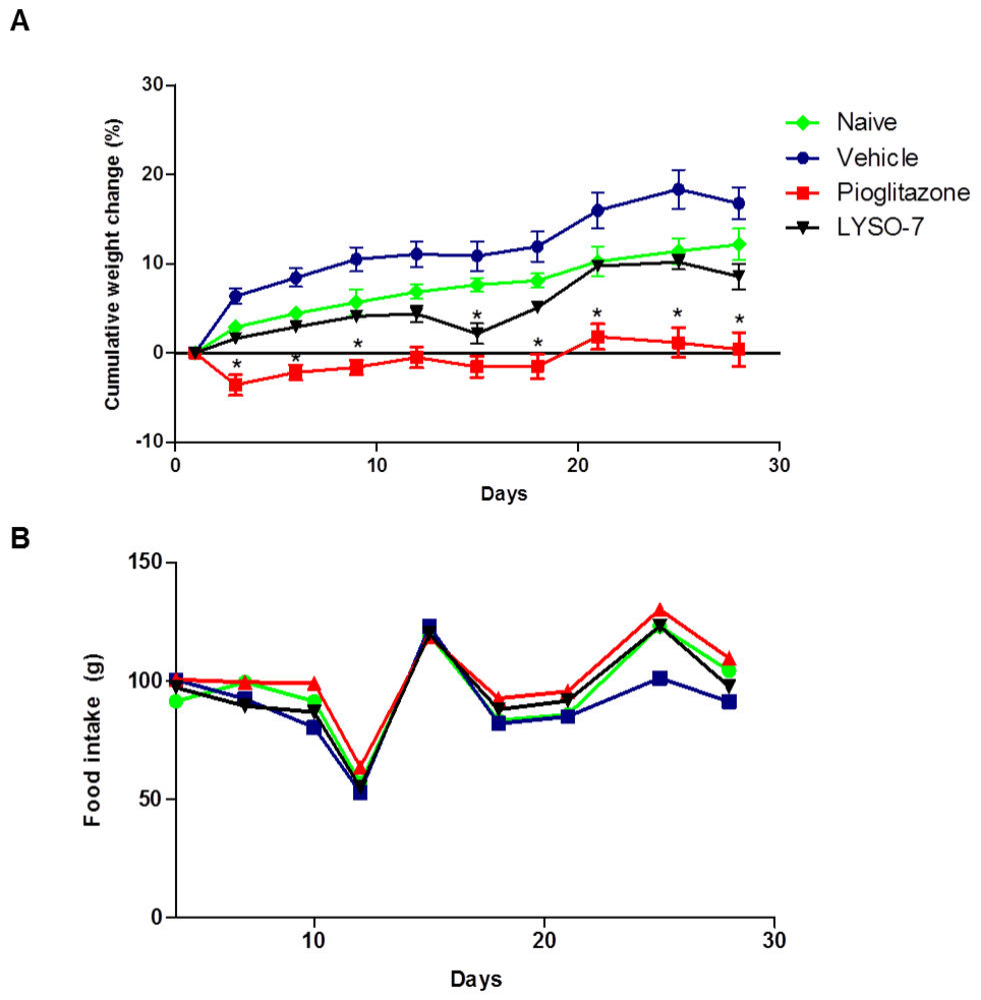
Effects of chronic LYSO-7 treatment on body weight and food intake. Male Swiss mice were treated with CMC (vehicle), pioglitazone or LYSO-7, p.o., once a daily for 28 days. (A) cumulative weight change (%); (B) food intake. Results are expressed as mean±SEM of 5 animals in each group. Statistical analysis was performed using ANOVA followed by Tukey’s test. *P<0.05 vs. vehicle.

In order to evaluate a possible adverse effect of LYSO-7, changes in body weight were evaluated for 28 days. The data show that LYSO-7 treatment did not alter the cumulative weight change or food intake. In contrast, pioglitazone treatment evoked significant weight loss, which was not dependent on impaired feed intake (Figure 8).

## Discussion and Conclusions

The effectiveness of preventive and therapeutic approaches for gastric ulcers has been limited to one pathway, i.e. proton pump inhibition, and the adverse effects of drugs [42]. Using an acute experimental model of gastric lesions, we show here that a indole-thiazolidine molecule, a PPAR pan-agonist and COX inhibitor named LYSO-7, does not affect gastric secretion, but causes cytoprotection by inhibiting neutrophil influx into the injured area and by maintaining blood flow in the gastric microcirculatory network. The latter effect is mediated by NO, which seems to be produced by eNOS*.*

The thiazolidine-2,4-dione region of the thiazolidione molecule binds to the retinoid X receptor (RXR) coupled to PPARs to form heterodimeric complexes, which then bind to the peroxisome proliferator response element (PPRE) gene promoter, leading to the regulation of gene transcription [43,44]. Although LYSO-7 maintains the thiazolidine-2,4-dione group, it is an indole-substituted properly synthesized to also display inhibitory activity against COX [33]. *In vitro* studies had already shown the PPAR pan-agonist activity of LYSO-7 [33], and here we confirm that the activity is maintained *in vivo*, as levels of PPARγ gene and protein expression were enhanced by LYSO-7 treatment. It is noteworthy that the expression of PPAR is a known end-point of PPAR activation [16]; however, the effect of a PPAR pan-agonist on gastric tissue has been shown here for the first time. Furthermore, we have shown that the cytoprotective effect of LYSO-7 is dependent on PPARγ, as the *in vivo* antagonism of the receptor by GW9962 abolished the inhibitory action of LYSO-7 in Et/HCl-induced ulcers. These data corroborate the notion that the γ isoform seems to be the main class of PPAR in gastric tissue [27–31]. It is worth mentioning that GW9962 has been previously used to determine the PPAR agonistic activity of newly synthesized compounds and to clarify the mechanisms of action of PPARγ [45–49].

Neutrophil influx has been observed in several models of gastric ulcers, and they have been thought to act as an inducer of the harmful process [50,51]. The participation of neutrophils in acute Et/HCl-induced gastric lesions in mice was shown here, as they rapidly accumulated in the injured tissue and *in vivo* neutrophil depletion significantly reduced the injured area. Together, these data corroborate the idea that inhibition of neutrophil recruitment may be a target for anti-gastric ulcer therapy [52,53], and that this can be modulated by LYSO-7 treatment.

The role of PPAR activation on neutrophil influx has been shown in different models of inflammation, and the majority of them show an inhibitory effect on the process [19,21,54]. The mechanisms involve the direct inhibition of leukocyte-endothelial interactions and chemotaxis [55,56] or impaired chemotactic mediator secretion [57–59]. Our data show, for the first time, that a PPAR agonist affects the trafficking of neutrophils from the bone marrow, as gastric-injured mice pre-treated with LYSO-7 presented higher and lower numbers of neutrophils in the bone marrow and blood, respectively. Our previous results indicate that LYSO-7 may act directly on the locomotory functions of neutrophils. N-formyl-l-methionyl-l-leucyl-l-phenylalanine (fMLP)-induced leukocyte-endothelial interactions in the mesenteric microcirculation are impaired in LYSO-7 treated rats, depending on reduced gene and protein expression of the CD62L and CD18 adhesion molecules by neutrophils (Farsky et al., personal communication). The results obtained in the present study contribute to this evidence, as the inhibitory effect on neutrophil trafficking was not dependent on NO mediation. The reduced neutrophil influx into gastric lesion caused by LYSO-7 was not modified by *in vivo* L-NAME treatment.

In contrast, maintenance of the surface mucosal microcirculatory blood flow by LYSO-7 treatment occurred via NO mediation, and seemed to be dependent on reduced and increased protein expression of iNOS and eNOS, respectively. A beneficial role of NO on gastric ulcers has been shown, as the *in vivo* blockade of both eNOS and iNOS favors the development of gastric lesions and treatments with NO donors heal lesions [60–62]. Although it has been shown that enhanced activities of iNOS and eNOS in arthritic rats are responsible for the aggravation of cold-resistant stress-induced gastric lesions [61], most studies have demonstrated that a shift in the eNOS/iNOS balance protects gastric tissue [13,62,63]. These data are corroborated here, as LYSO-7 promoted a shift in eNOS/iNOS expressed proteins in gastric tissue. It has been shown that PPAR selective agonists favor the shift of the eNOS/iNOS balance in renal tissue after ischemia–reperfusion injury, in endothelial cells after glycation-end product activation, and during vasoconstriction in mesentery vessels [64–67]. Nevertheless, it seems that activation of the three isoforms of PPAR are involved in NO-mediated gastroprotection, as the shift in eNOS/iNOS expression and the reversion of glutathione peroxidase and reductase levels in the damaged stomach were not detected after equivalent treatment with pioglitazone, a selective PPARγ agonist (data not shown). Further assays are being carried out to confirm this hypothesis.

The development of PPAR pan-agonists has been focused on the reduction of adverse effects, as they act as partial agonists of two or more isoforms of the receptor [23,68]. Although the data are preliminary, LYSO-7 did not modify body weight, which was reduced by equivalent treatment with pioglitazone. In addition, no alterations to biochemical and hematological parameters or on the morphology of the heart, spleen, liver, lung and kidney were observed (data not shown). Considering that the therapeutic efficacy of PPAR agonists is a worrying challenge [68], additional toxicity and kinetics assays were performed to assure the efficacy of LYSO-7.

In summary, we have shown the *in vivo* actions of LYSO-7 on the mechanisms of gastric ulcer pathogenesis. We have also demonstrated that inhibition of neutrophil migration and a shift in the eNOS/iNOS balance completely abrogate gastric tissue lesions. Based on the mechanism elucidated here, LYSO-7 may be employed to treat neutrophil-mediated diseases and to reestablish damaged microcirculatory networks.
